# When Omental Infarction Imitates Appendicitis: Advancing Diagnosis with POCUS

**DOI:** 10.24908/pocusj.v10i02.19337

**Published:** 2025-11-17

**Authors:** David J. McCreary, Iain Fraser, Nigel Chan

**Affiliations:** 1 Paediatric Emergency Department, Sunderland Royal Hospital, Kayll Road, Sunderland, SR4 7TP, UK; 2 Paediatric Emergency Department, Royal Hospital for Children, Glasgow, NHS Greater Glasgow & Clyde

**Keywords:** POCUS, Appendicitis, Omentum, Omental infarction, Free fluid

## Abstract

A previously healthy 12-year-old boy presented to the paediatric emergency department on three occasions in the space of four days with progressive right iliac fossa pain. His presentation appeared consistent with appendicitis; however, blood tests remained normal at each presentation. Point of care ultrasound (POCUS) revealed significant inflammatory changes in the right lower quadrant along with free fluid. Due to worsening pain and advancing findings on POCUS, the patient underwent a diagnostic laparoscopy which identified a normal appendix but had features consistent with omental infarction. Omental infarction is a rare condition that can mimic appendicitis, with few recorded cases in the literature. This is the first documented case of POCUS being utilised in the evaluation of a child with omental infarction. While the patient's diagnosis was less common than initially suspected, POCUS played a crucial role in guiding timely and effective patient care, highlighting its value in clinical decision-making However, there are indications of lower POCUS sensitivity to fluid overload with a full peritoneum.

## Case history

**Figure 1.**
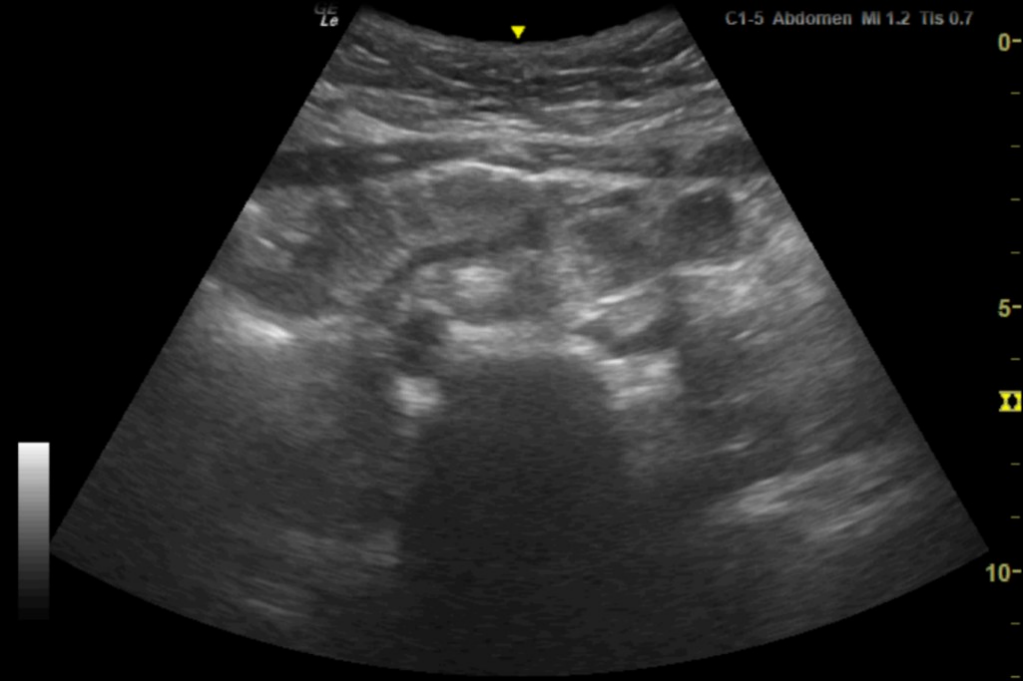
Point of care ultrasound (POCUS) image of lower abdomen in transverse orientation using the 1-5 MHz curvilinear probe. There is a suspicion of a small amount of free fluid in the right lower quadrant.

**Figure 2.**
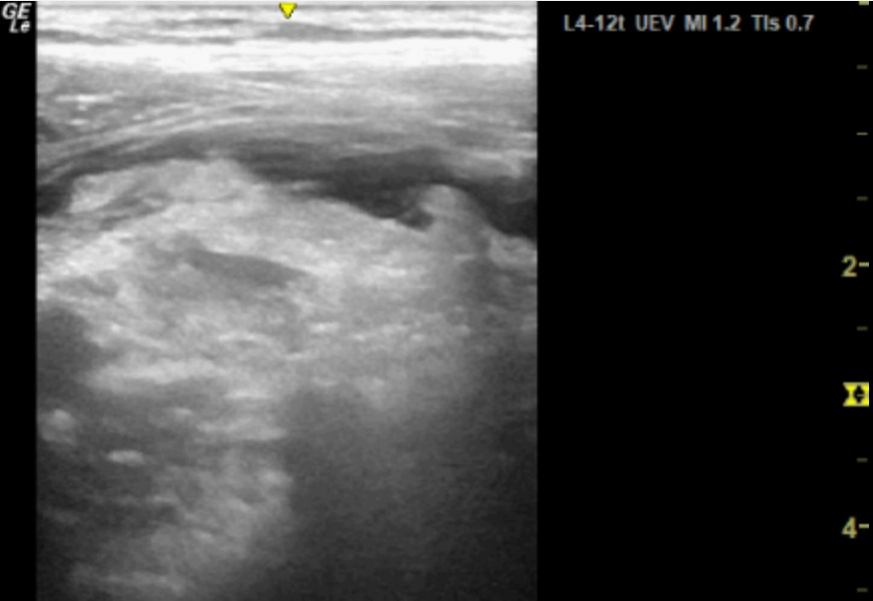
Point of care ultrasound (POCUS) image of right iliac fossa using the high frequency 12 MHz linear probe demonstrating significant hyperinflammatory changes in the right lower quadrant and a moderate amount of localised free fluid. This examination was associated with a significant degree of sonographic tenderness.

**Figure 3.**
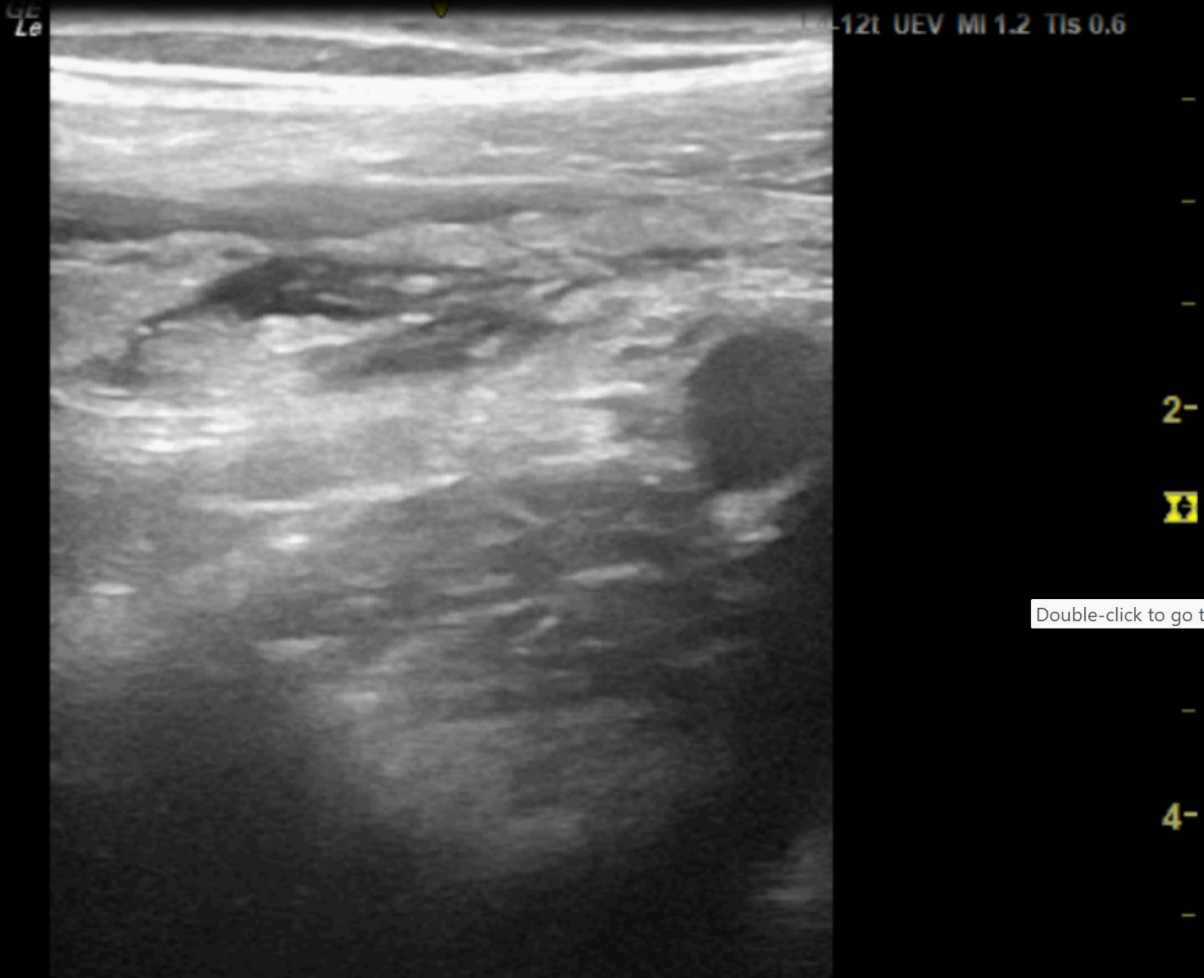
Point of care ultrasound (POCUS) image of right iliac fossa using the high frequency 12 MHz linear probe. This shows a moderate degree of echogenic inflammatory changes felt to be consistent with fat wrapping. There is small region of localised free fluid within this.

**Figure 4.**
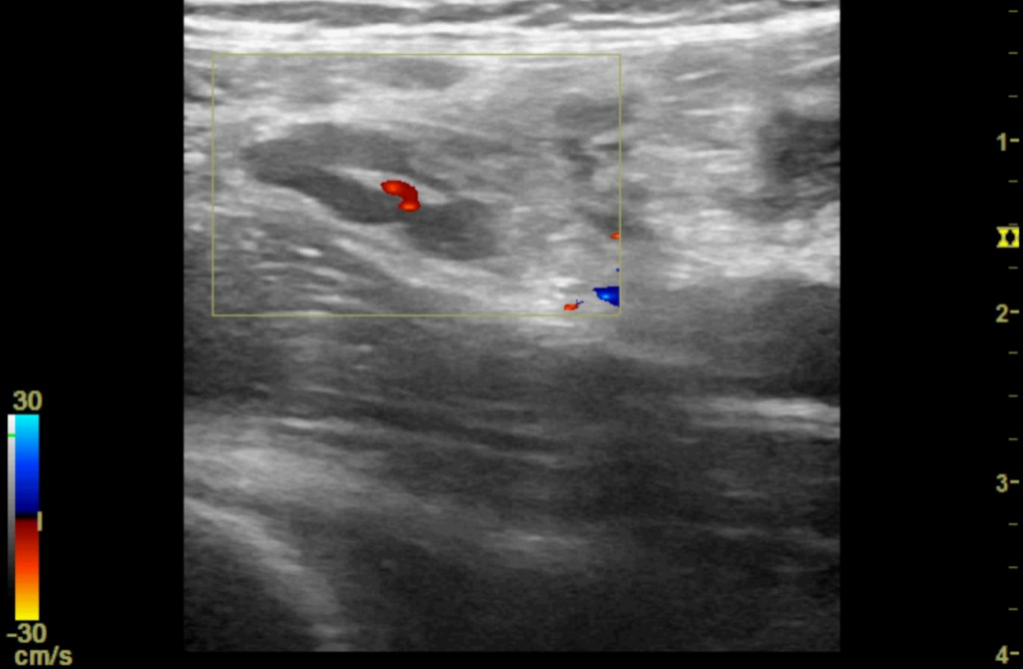
Point of care ultrasound (POCUS) image of right iliac fossa using the high frequency 12 MHz linear probe. This shows a small anechoic region felt to be consistent with a pocket of free fluid. There is degree of colour Doppler enhancement within the central component.

A previously healthy 12-year-old boy presented to the paediatric emergency department having experienced abdominal pain for the previous 24 hours. His pain was described as sharp and of relatively sudden onset in the right iliac fossa with no preceding migratory pain. He had one episode of vomiting overnight but with no diarrhoea. He described anorexia and feeling warm, though no fever was recorded. His vital signs were normal, and he appeared comfortable at rest, though had an obvious altered gait when walking to the examination room. Examination revealed discomfort in the right iliac fossa, but no rebound tenderness. Psoas sign was positive, however Rovsing's sign was negative. His scrotal examination and urine dip were also unremarkable. One of the authors undertook a POCUS examination of his abdomen which demonstrated significant hyperinflammatory changes in the right lower quadrant and a moderate amount of localised free fluid, however, the appendix was not visualised (See Figures 1-4). His blood tests were as follows: White Blood Cell Count (WBC) 7.11 x 109, neutrophils 3.87 x109, C-Reactive Protein (CRP) 2.4 mg/L. He was reviewed by the surgical team who felt his clinical appearances and blood tests were not consistent with a surgical cause and he was discharged home.

The boy re-presented to the department 3 days later with progressive pain. He continued to have a significantly altered gait and his mother described that he had been “unable to move” due to the pain and thus had not been sent to school. He had not developed vomiting, fever, or any other new symptoms. On examination, his abdomen was soft, but he remained tender in the right iliac fossa. Psoas sign was positive and Rovsing's sign was negative.

**Figure 5.**
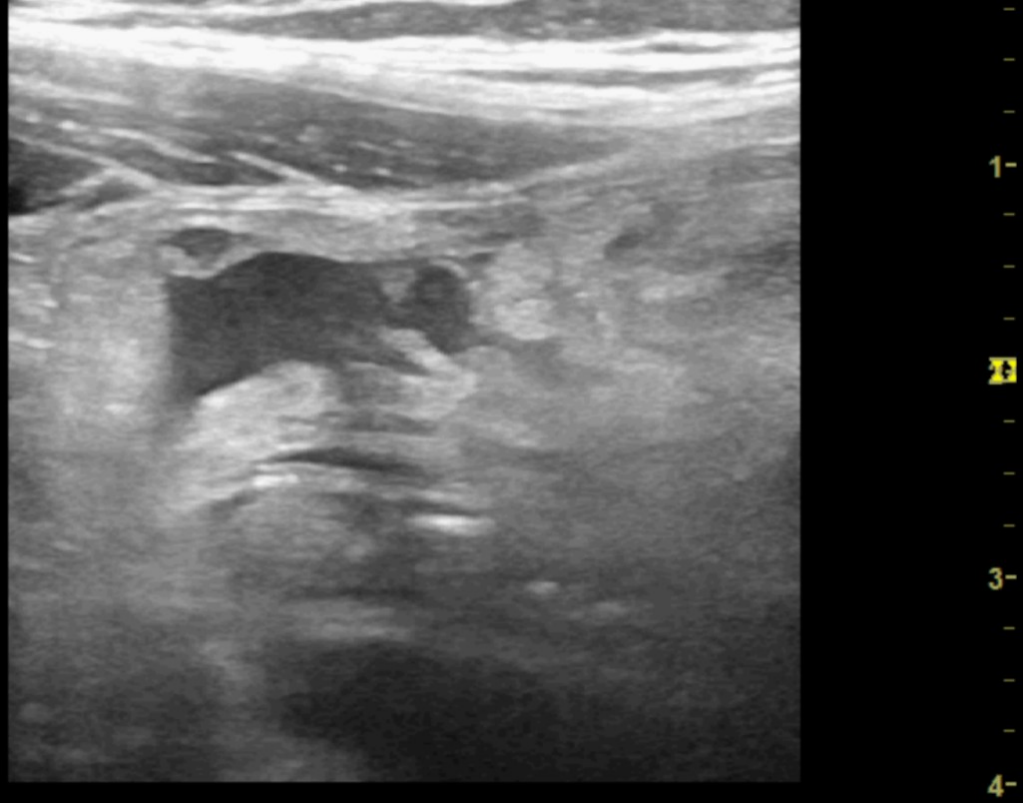
Point of care ultrasound (POCUS) image of right iliac fossa using the high frequency 12MHz linear probe. This shows significant hyperechoic change along with a pocket of free fluid.

**Figure 6.**
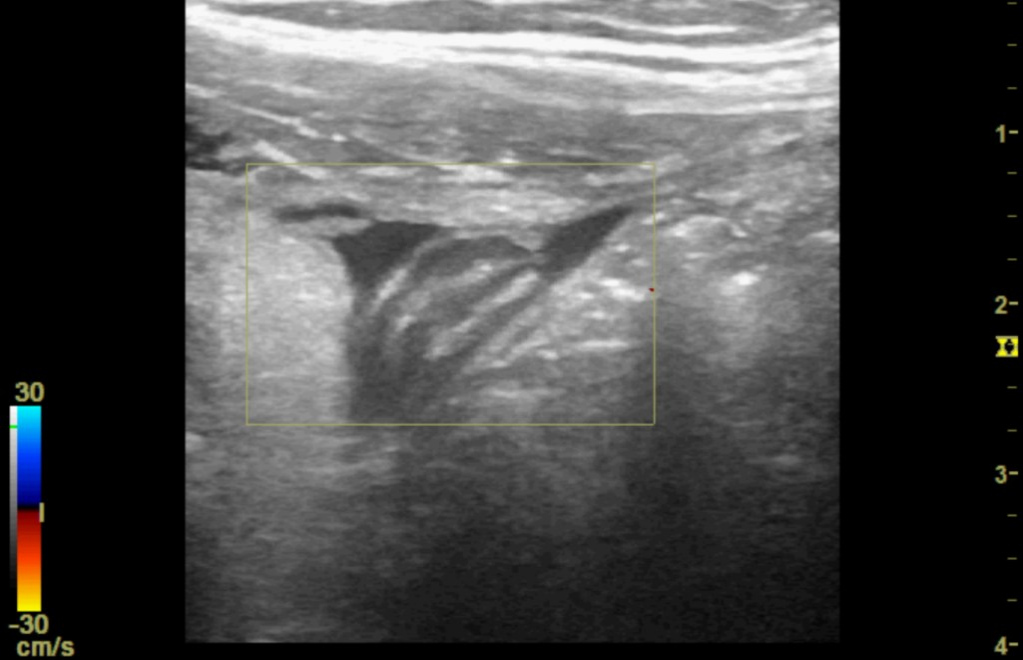
Point of care ultrasound (POCUS) image of right iliac fossa using the high frequency 12 MHz linear probe. This shows further inflammatory change along with what appears to be a blind ending, non-peristalsing tubular structure surrounded by localised free fluid but without evidence of colour Doppler enhancement.

**Figure 7.**
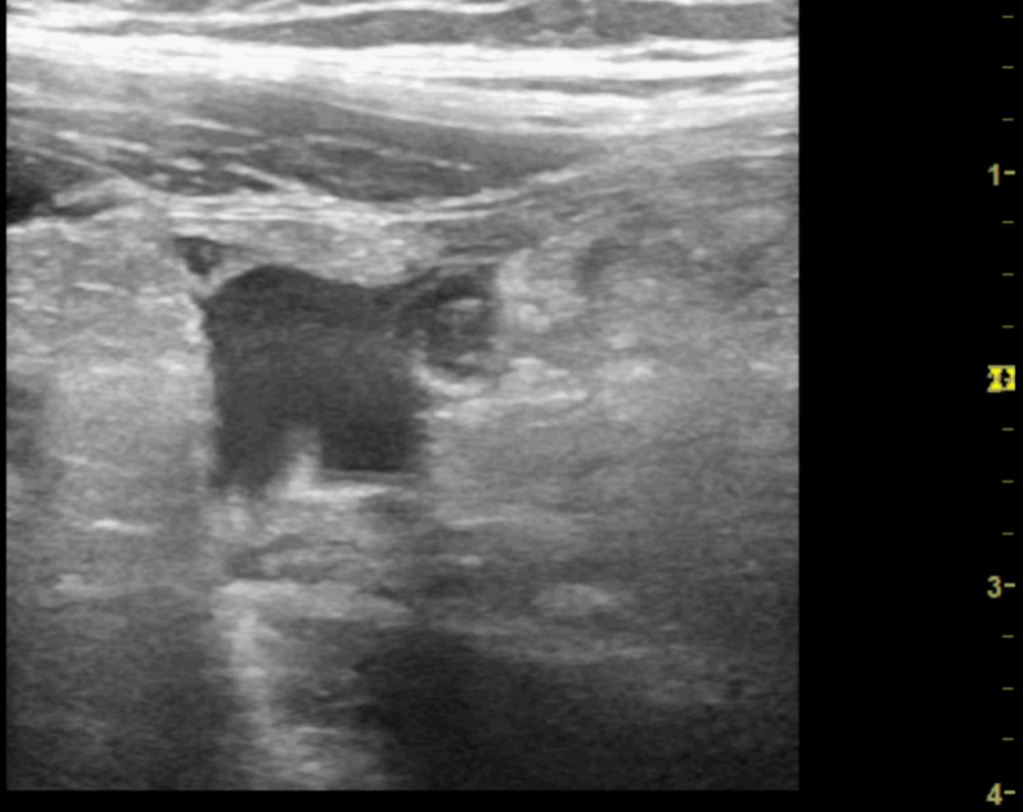
Point of care ultrasound (POCUS) image of right iliac fossa using the high frequency 12 MHz linear probe. This shows progression of the echogenic inflammatory change and a greater degree of free fluid. A tubular structure with characteristics consistent with the appendix can be seen in a transverse orientation.

**Figure 8.**
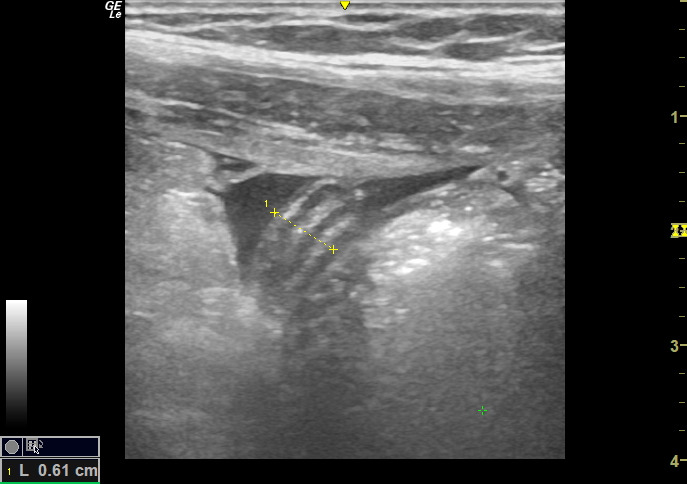
Point of care ultrasound (POCUS) image of right iliac fossa using the high frequency 12 MHz linear probe. Visualised within a pocket of free fluid is a tubular structure with wall signature and mucosal characteristics consistent with the appendix measuring 6.1 mm in diameter.

His vital signs remained stable and repeat blood tests were as follows: WBC 7.92 x 109, neutrophils 3.59 x 109, CRP 5.2 mg/L. Repeat POCUS was performed which demonstrated progressive hyperinflammatory, echogenic changes and a moderate amount of localised free fluid. Within this free fluid there appeared to be a blind ending tubular structure without peristalsis measuring 0.61 cm in diameter. While there was no colour Doppler enhancement, this area was consistent with significant sonographic tenderness (See Figures 5-8).

**Figure 9.**
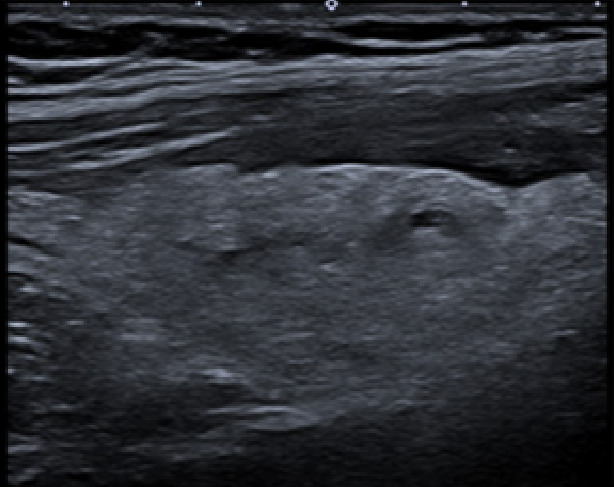
Radiology-performed ultrasound of the right iliac fossa with a high frequency linear probe demonstrating significant hyperinflammatory changes. When taking clinical presentation into account, this report concluded that appearances were consistent with likely appendicitis with perforation.

He was again reviewed by the surgical team. They felt that in view of his right iliac fossa being only mildly tender and his normal blood tests that he did not warrant diagnostic laparoscopy. The findings on POCUS were described to the surgical team, at which point recommendation was made for a radiology-performed ultrasound to be conducted. This could only be arranged for the following day. When the patient returned to the paediatric emergency department the next day, a radiology-performed ultrasound took place (See Figure 9). The report was as follows: “extensive echogenicity with surrounding fluid in the right iliac fossa suggestive of an inflammatory process, in the presence of a non-visualised appendix the possibility of perforated appendix cannot be ruled out.” Blood tests taken on this occasion were as follows: WBC 6.58 x 109, neutrophils 3.32 x 109, CRP 3.3 mg/L.

The patient subsequently underwent a laparoscopy which identified a normal appendix but an area of thickened, white omentum consistent with omental infarction.

## Introduction

Omental infarction is a rare cause of abdominal pain in children and usually presents with acute abdominal pain [1,2]. It is more common in children who are overweight [1]. Pain from omental infarction typically affects the right side of the abdomen, attributed to right segmental infarction from blood vessels in the omentum being more susceptible to torsion because of their longer size and higher mobility [2,3].

Previously, omental infarcts were most often detected intra-operatively during surgery for presumed appendicitis or other causes of acute abdomen pain, but the increased use of imaging, particularly ultrasound and Computed Tomography (CT), has led to increases in preoperative diagnosis of omental infarction [4,6]. Omental infarcts are found in <1% of patients undergoing surgery for suspected appendicitis [5]. Having both acute appendicitis and omental infarction is extremely rare, with only two cases reported in the literature [3].

## Literature review

To our knowledge, no published literature exists pertaining to the use of POCUS as part of the evaluation of omental infarction in adults or in a child. Omental infarction can mimic common abdominal pathologies such as appendicitis and cholecystitis. A 2018 case report by Lindley and Peyser discussed a 42-year-old male who presented with right-sided abdominal pain [8]. He was suspected of having cholecystitis, and later underwent a radiology-performed ultrasound which found normal anatomy in the right upper quadrant but some trace free fluid in the right iliac fossa. This prompted a CT scan to be undertaken which showed a normal appendix but tissue stranding adjacent to the appendix. A subsequent laparoscopy was performed due to onset of fever and worsening pain. This highlighted an inflamed and necrotic greater omentum with a normal appendix.

Kozłowski, Piotrowska and Giżewska-Kacprzak [6] detailed a case report of a 9-year-old girl who had presented to hospital with epigastric pain. She had evidence of faecal loading of the colon but disproportionate levels of pain with elevated inflammatory markers. Abdominal ultrasound showed mesenteric adipose tissue with increased echogenicity, trace fluid in the rectouterine pouch and no visualised appendix. This prompted subsequent CT imaging, which showed fat stranding in the right epigastrium with a normal appendix. This patient was managed conservatively with antibiotics and analgesia.

In a report from Grattan-Smith, Blews and Brand, sonographic imaging alone did not find any abnormal findings in two children who subsequently underwent a laparoscopy where omental infarction was later diagnosed [10]. Four other patients with abnormal sonographic findings subsequently underwent CT imaging where heterogenous fatty masses were found anterior to the ascending colon. Laparoscopic evaluation later identified omental infarction.

### Mimics of appendicitis on POCUS

There are many conditions that present similarly to appendicitis. They can cause sonographic appearances of periappendiceal inflammatory changes with secondary thickening of the appendix which can subsequently be mistaken for appendicitis. Examples include terminal ileitis secondary to Crohn's disease, tuboovarian abscess, typhilitis and sigmoid diverticulitis [7,8].

Lymphoid hyperplasia can result in a non-compressible appendix greater than 6 mm in diameter which can be mistaken for appendicitis. The sonographic appearance of lymphoid hyperplasia has been described as an enlargement of the hypoechoic lamina propria to a thickness of greater than 8 mm [9]. The lamina propria forms the middle layer of the gastrointestinal mucosa, including in the appendix. It contains lymphoid follicles which can hypertrophy in response to inflammation—such as from viral gastroenteritis or mesenteric adenitis, which are common conditions in the paediatric population that can also present similarly to appendicitis [10].

A study by Aydin et al. suggested that the presence of local fluid collection in the periappendiceal area and a lamina propria thickness of <1 mm are the most useful sonographic parameters for distinguishing between appendicitis and lymphoid hyperplasia [11]. Similarly, Xu et al. suggested that true positive diagnoses of appendicitis are more accurately identified with the presence of at least two additional sonographic findings of periappendiceal fluid, hyperechoic fat, or mural hyperaemia [12].

Meckel's diverticulum is the most common congenital abnormality of the gastrointestinal tract with an estimated prevalence of 2%. It is most often located near the ileocaecal valve in the right lower quadrant near the appendix. Because of this proximity, if it is inflamed or torsed it can clinically and sonographically mimic acute appendicitis. An inflamed Meckel's diverticulum can appear very similar to appendicitis on ultrasound with a thick-walled, blind-ending structure and associated hyperechoic inflammatory changes near the caecum [13]. Choi et al. presented a case report of a 38-year-old female who presented with right lower quadrant abdominal pain. POCUS showed a 9 mm tubular, non-peristalsing structure superficial to the iliac vessels with a target appearance on short axis which was interpreted as appendicitis. The patient was subsequently found to have a normal appendix but a torsed Meckel's diverticulum intra-operatively [14].

Shavor et al. described a 17-year-old male who presented with right lower quadrant pain. POCUS showed a non-peristalsing, non-compressible tubular structure adjacent to the iliac vessels with a hyperechoic structure with acoustic shadowing noted within the lumen. This was initially considered to be an appendicolith, but CT demonstrated an 11 mm obstructing ureteral stone [15].

Omental infarction is another pathology that in may mimic appendicitis. Esposito et al. highlighted key sonographic findings suggestive of omental infarction often as a hyperechoic, non-compressible mass beneath the anterior abdominal with a heterogenous echotexture [1]. Colour or power Doppler has been described as a useful adjunct, when used to demonstrate reduced or absent flow within the region of omentum. This differs from appendicitis where enhanced colour flow is often present.

## Discussion

This case illustrated the central role of POCUS in highlighting evidence of abnormalities indicative of lower quadrant pathology that warranted surgical attention.

Unlike previously reported cases of omental infarction where diagnostic pathways typically involved radiology-performed ultrasound and CT, our case showed how POCUS was incorporated into the initial workup. This was able to be easily and safely repeated, allowing for progression of the pathological process to be closely monitored.

The value of POCUS is particularly significant in settings where access to specialist imaging services is limited. Mathers et al. surveyed imaging services across England, Wales and Scotland which highlighted a paucity of trained paediatric radiologists [20]. This is not a unique workforce gap but is mirrored in other healthcare systems globally, as highlighted by Stringer and Pledger [16]. In this context, POCUS therefore offers a pragmatic, readily accessible tool to support timely and accurate clinical decision-making.

The consensus as to whether operative versus conservative management is recommended for omental infarction has not yet been reached. Recent studies suggest that an initial trial of conservative management, including analgesia and close monitoring, is appropriate for stable patients with a confirmed diagnosis via imaging. A systematic review including 146 patients by Medina-Gallardo et al. found that conservative treatment was effective in most patients, but those undergoing surgery typically had shorter in-hospital stays [22]. A 10-year case series carried out by Diab et al. concluded that a trial of conservative management should be recommended for 24-48 hours before considering surgical treatment in refractory cases [23]. Ultimately, the decision for conservative or surgical management is individualised, accounting for the patient's clinical presentation, imaging findings and response to conservative management if instituted.

This case highlights the benefits of POCUS as a fast, effective, and easily repeatable imaging modality that avoids exposing a patient to potential harm from ionising radiation. POCUS has been demonstrated to be useful in the initial assessment children and young people presenting with undifferentiated abdominal and pelvic pain [17,18]. It has further shown to decrease radiology department ultrasound requests across a range of hospital settings [19,20]. These demonstrations may have positive implications from both a cost benefit perspective as well as for improving departmental flow.

## Conclusion

Our case demonstrated the role of POCUS in the evaluation of a child with undifferentiated right-sided abdominal pain, where appendicitis had clinically been suspected, but the final diagnosis was omental infarction. Despite normal inflammatory markers and non-specific clinical signs, POCUS identified progressive inflammatory changes in the right iliac fossa that prompted further investigation and ultimately led to diagnostic laparoscopy. This approach avoided the need for ionising radiation. In an era of increasing demands on imaging services and limited access to paediatric radiologists, POCUS remains a pragmatic, repeatable and low-risk diagnostic tool in the hands of appropriately trained clinicians.

As this case demonstrated, incorporating POCUS into the assessment of abdominal pain can facilitate earlier recognition of rare pathologies and support informed decision-making. In this case, the patient's family expressed their gratitude to the clinicians, recognising their advocacy for their child through the effective use of POCUS. Importantly, this case also highlights that not all right lower quadrant pain or abnormalities detected on POCUS should be assumed to be appendicitis. Furthermore, it suggests a potential role for POCUS in the diagnostic pathway for omental infarction, particularly as an initial imaging modality. Compared to radiology-performed ultrasound, POCUS offers the advantage of being rapidly available and easily repeatable at regular intervals, thereby enabling definitive, expedited patient management.
